# Editorial: Discovery and Development of Drugs for Neglected Diseases: Chagas Disease, Human African Trypanosomiasis, and Leishmaniasis

**DOI:** 10.3389/fchem.2021.775327

**Published:** 2021-10-07

**Authors:** Gildardo Rivera, Navin B. Patel, Debasish Bandyopadhyay

**Affiliations:** ^1^ Laboratorio de Biotecnología Farmacéutica, Centro de Biotecnología Genómica, Instituto Politécnico Nacional, Reynosa, Mexico; ^2^ Department of Chemistry, Veer Narmad South Gujarat University, Surat, India; ^3^ Department of Chemistry, University of Texas Rio Grande Valley, Edinburg, TX, United States; ^4^ School of Earth Environment and Marine Sciences (SEEMS), University of Texas Rio Grande Valley, Edinburg, TX, United States

**Keywords:** trypanocidal, leishmanicidal, trypanosomiasis, neglected tropical diseases, therapeutics, drug development

**FIGURE 1:**
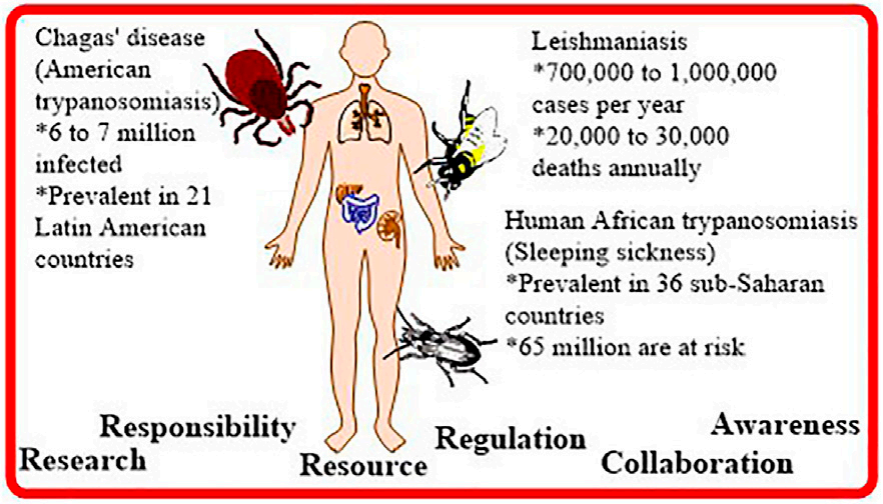
Global scenario of three neglected tropical diseases: Chagas disease (American trypanosomiasis), Human African trypanosomiasis (HAT), and Leishmaniasis.

Twenty tropical diseases have been listed by the World Health Organization (WHO) as *Neglected Tropical Diseases* (NTDs). These tropical diseases are called “neglected” for three primary reasons: 1) These diseases are widespread worldwide among the economically weaker (neglected?) section of the society; 2) Although the total number of mortality, morbidity, disability, and health disparity caused annually by the NTDs is more than that attributed by the so-called *elite* diseases like cancer, diabetes, human immunodeficiency virus/acquired immunodeficiency syndrome (HIV/AIDS), cardio- or neurological diseases but NTD patients do not receive comparable attention or treatment opportunities either from the governments or healthcare professionals or non-governmental organizations (NGOs); 3) The scope of drug development research in this field is minimal due to insufficient (negligible) funding, and pharma giants are not interested in developing effective drugs for NTDs on time, due to insignificant profit. It is highly challenging to develop new and novel drugs to combat these tropical diseases to upthrust the lifespan and lifestyle of the socioeconomically deprived people affected by NTDs (Figure 1). The major goal of this research topic is to shed light on the global scenario (current status of the ailments, treatment options, and recent drug development efforts) of three major NTDs, *viz*. Chagas disease (American trypanosomiasis), Human African trypanosomiasis (HAT), and Leishmaniasis. This research topic contains three reviews and seven research articles.

Mother Nature can be an excellent source of leishmanicidal compounds. An in-depth review summarizing the leishmanicidal activity of various crude extracts and isolated compounds against promastigote and amastigote (intracellular and axenic) forms has been published (Gervazoni et al.). The leishmanicidal activity of different classes of compounds such as alkaloids, terpenoids, flavonoids, coumarins, quinones, and their respective biosynthetic pathways, published between 2000–2020, has been counted in. Mechanisms of action of various natural compounds (if known) have also been discussed in this review.

Bijlmakers specified the limitation of effective drugs for treating trypanocidal and leishmanicidal infections. In treating trypanosomatid diseases, the author reviewed the protozoal ubiquitin-proteasome system as potential therapeutic targets (primarily UBA1 and ubiquitin-like enzymes). This review discussed the mechanisms of action of a series of ubiquitination inhibitors.

Brindha et al. reviewed the mechanisms of action of the target-based trypanocidal and leishmanicidal pharmaceutics and druglike molecules. A few significant biomolecular targets such as trypanothione reductase, cathepsin l-cysteine protease (cruzain), CYP51 (sterol 14-ademethylase), kinetoplastid proteasome, heme peroxidation, have been discussed in detail. This review also includes a discussion on nano-formulation of the therapeutics.

The *Leishmania infantum* arginase (LiARG) was considered as a drug target for antileishmanial evaluation, and a series of chalcone derivatives were screened (Garcia et al.). In this study, three chalcone derivatives were identified as LiARG inhibitors. Moreover, three chalcone derivatives demonstrated activity against the promastigote and intracellular amastigote forms of *Leishmania infantum*. The authors proposed the presence of a nitro group at the *para*-position on chalcone “B” ring as an essential factor to induce leishmanicidal activity.

The heat shock protein 90 (Hsp90) inhibitors such as 17-dimethylaminoethylamino-17-demethoxygeldanamycin (17-DMAG) could be a therapeutic option for treating cutaneous leishmaniasis. Cruz et al. reported developing 17-DMAG-containing nanoparticles using poly(lactic-co-glycolic acid) *via* a double emulsion procedure. The nanoparticles were internalized by macrophages and accumulated in the cell cytoplasm. The authors evaluated the release kinetics of 17-DMAG from the nanoparticle.

Chemical modification of medicinally privileged molecules targeting more potent and/or selective molecules is ongoing in drug discovery research. Sijm et al. successfully conducted chemical modification of one of their previously reported trypanocidal molecules with particular attention to reduce the cLogP values of their newly synthesized molecules with similar potency. It is expected that the newly synthesized *N*-substituted dihydropyrazolone derivatives should demonstrate better drug metabolism and pharmacokinetics (DMPK) properties (Sijm et al.). The authors have studied the qualitative structure-activity relationship (SAR) by diversifying the substituent on the nitrogen atom adjacent to the carbonyl group in the dihydropyrazolone core.

The small molecule TbrPDEB1 inhibitors can play an important role against the protozoan *T. brucei*. Thus, a series of small molecule tetrahydrophthalazinone derivatives have been designed through structure-guided virtual screening (Heuvel et al.). A multi-step sequence consisting of mesylation-Friedel-Crafts acylation-Diels-Alder reaction-heterocyclization-demesylation was used to synthesize the guaiacol-bearing tetrahydrophthalazinone derivatives. Therefore, the phenolic-OH group was explored to synthesize a series of corresponding diaryl/heteroaryl ether derivatives. The compounds showed selectivity while compared to MRC-5 cell lines.

Cu(II)-mediated 1,3-dipolar alkyne-azide cycloaddition strategy was followed to synthesize 16 triazolyl proline derivatives (Fargnoli et al.). The authors evaluated the end-products as l-proline transport inhibitors against *Trypanosoma cruzi* epimastigotes, and cytotoxicity was measured in VERO cell lines. This study identified the 1, 2, 3-triazole as an appropriate linker for the transport inhibitors and effectively validated l-proline uptake blockers as trypanocidal agents.

Amphotericin B is a natural polyene compound that belongs to the WHO’s list of essential medicines. Gedda et al. developed a nanostructure-based method for targeted delivery of Amphotericin B. The f-Comp-AmB nanocomposite demonstrated multi-fold better leishmanicidal activity than two previously reported nanocomposites of the drug and the drug itself against the intracellular amastigotes of *L. donovani* in the J774A.1 cell lines. In addition, the f-Comp-AmB composite showed lower cytotoxicity than two other nanocomposites of Amphotericin B.

Environmental protection is one of the significant criteria in modern drug discovery research. Rock et al. reported an ultrasound-assisted on-water synthesis of benzopyrazines without using any catalyst/support/additive. A series of eleven benzopyrazines were synthesized and evaluated *in vitro*. One benzopyrazine demonstrated comparable trypanocidal and leishmanicidal activities with commercial drugs (Rock et al.). Two other benzopyrazines showed moderate leishmanicidal activity against *L. mexicana* (M378) strain. The authors hypothesized the inhibition of four enzymes as biomolecular targets and validated the hypothesis by *in silico* and *in vitro* evaluations.

In a timely manner, this research topic presents a concise account of the current status and drug discovery approaches related to three neglected tropical diseases: Chagas disease, HAT, and leishmaniasis. Appropriate actions should be taken to protect millions of people worldwide who are struggling with a fatality, disability, disruption of social life, and seclusion.

